# Disparities in Breast Cancer Screening, Diagnosis, and Outcomes Among Vietnamese American Women: A Systematic Review

**DOI:** 10.1002/jso.70164

**Published:** 2026-01-14

**Authors:** Antoinette T. Nguyen, Emily D. Duckworth, Rena A. Li, Robert D. Galiano

**Affiliations:** ^1^ School of Medicine and Dentistry University of Rochester Rochester NY USA; ^2^ School of Medicine Greenville University of South Carolina Greenville South Carolina USA; ^3^ Northwestern University Feinberg School of Medicine Chicago Illinois USA

**Keywords:** breast cancer, cancer screening, healthcare disparities, systematic review, Vietnamese American

## Abstract

Breast cancer remains a leading cause of cancer‐related mortality among women globally. Vietnamese women experience unique challenges, including sociocultural, linguistic, and systemic barriers, contributing to disparities in screening utilization, late‐stage diagnoses, and treatment outcomes. Despite advances in early detection and care, inequities persist. A systematic review was conducted following PRISMA guidelines, with the protocol registered on PROSPERO. PubMed, Embase, and Scopus were searched for original studies published from 2000 to 2024 examining breast cancer screening, outcomes, molecular/genetic features, and disparities in Vietnamese populations. Narrative synthesis was employed due to heterogeneity in study metrics and methodologies. Forty‐one studies encompassing 39,324 Vietnamese participants (mean age 48.15 ± 7.48 years) were included. Social networks and acculturation positively influenced screening uptake, while systemic barriers such as language, cultural stigma, and lack of insurance deterred participation. Across included studies, mammography screening rates among Vietnamese women ranged widely from 26% to 83%, consistently lower than the U.S. national average of 81%, and lower than rates reported in many Asian American subgroups. Late‐stage diagnoses were prevalent, occurring in 32.9% of Vietnamese women, with foreign‐born Vietnamese women exhibiting higher mortality than U.S.‐born counterparts. Molecular studies revealed distinct tumor subtypes, including higher HER2‐positive and triple‐negative breast cancer rates. Interventions, including culturally tailored education and patient navigator programs, demonstrated success in addressing screening and care disparities. Vietnamese women face significant breast cancer disparities driven by sociocultural, systemic, and biological factors. Effective solutions require integrating culturally tailored solutions to promote equitable outcomes and reduce disparities in breast cancer care.

## 1 Introduction

Breast cancer remains the most frequently diagnosed cancer and a leading cause of cancer‐related mortality among women globally [1]. Despite significant advances in early detection and treatment that have improved survival rates, inequities in screening, diagnosis, and care continue to disproportionately affect ethnic minority populations [2, 3]. Vietnamese women, in particular, represent a unique group that faces distinct sociocultural, linguistic, and systemic barriers in breast cancer prevention and treatment [4]. Within the United States, studies have shown that Vietnamese women have lower rates of breast cancer screening, higher rates of late‐stage diagnoses, and limited access to culturally tailored healthcare interventions [5, 6, 7]. These challenges contribute to disparities in outcomes and highlight the urgent need for targeted solutions. In addition to sociocultural and systemic factors, biological differences in breast cancer characteristics among Vietnamese women have begun to emerge in the literature. Molecular and genetic studies reveal distinct tumor subtypes and biomarker expression profiles that may influence prognosis and therapeutic response in this population [8]. These findings underscore the importance of integrating biological insights with social and structural determinants of health to develop a more comprehensive understanding of the breast cancer burden in Vietnamese women.

This systematic review synthesizes evidence published between 2000 and 2024 to examine the multifaceted challenges faced by Vietnamese women in the context of breast cancer screening, diagnosis, treatment, and outcomes. Drawing from studies conducted across diverse contexts, this review explores breast cancer screening behaviors, disparities in access to care, biological and molecular characteristics of breast cancer, and the effectiveness of interventions tailored to this population. By analyzing these intersecting domains, this review aims to illuminate patterns and gaps in the current evidence base and provide actionable recommendations for addressing breast cancer disparities in Vietnamese women through culturally and contextually informed approaches.

## 2 Materials and Methods

**Figure 1:**
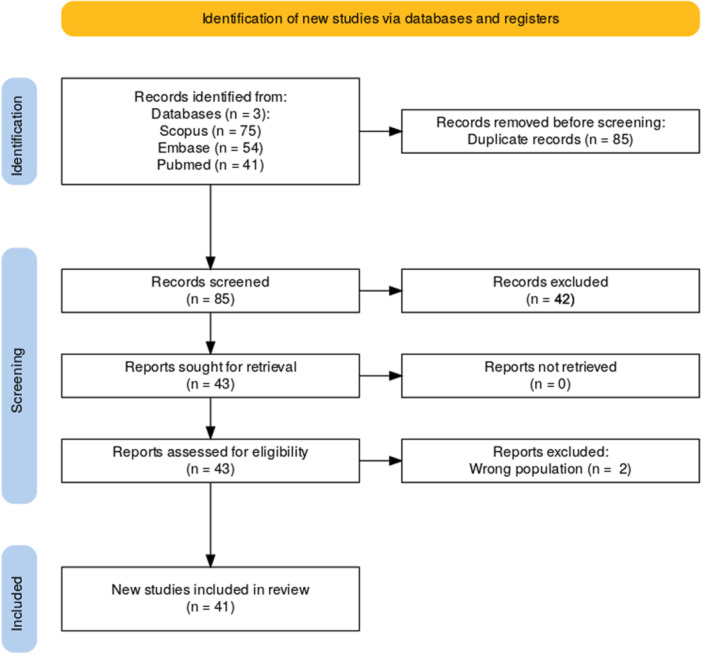
PRISMA Flow diagram of study selection (2000–2024).

This systematic review followed the Preferred Reporting Items for Systematic Reviews and Meta‐Analyses (PRISMA) guidelines to ensure a transparent and rigorous process (Figure 1). The protocol was registered with the International Prospective Register of Systematic Reviews (PROSPERO) (Registration ID: CRD42024654266) to predefine objectives, inclusion criteria, and methodologies, minimizing potential biases. A comprehensive literature search was conducted across PubMed, Embase, and Scopus databases to identify relevant studies published between January 1, 2000 and December 31, 2024. The search strategy utilized keywords and Medical Subject Headings (MeSH) terms related to “Vietnamese,” “breast cancer,” “screening,” “outcomes,” “interventions,” and “disparities.”

Inclusion criteria encompassed original studies that examined breast cancer screening, outcomes, molecular/genetic features, or disparities among Vietnamese populations. Only studies providing primary data, published in English, and offering separate findings for Vietnamese participants were included. Reviews, commentaries, editorials, and abstracts without accessible full text were excluded. Two reviewers independently screened titles and abstracts, followed by full‐text evaluation for eligibility, with disagreements resolved through discussion or consultation with a third reviewer. Data extraction was performed using a standardized form to capture key variables, including author(s), publication year, study design, sample size, Vietnamese participant demographics, breast cancer metrics, and statistical findings. Two reviewers extracted data independently to ensure accuracy and consistency, and discrepancies were resolved through consensus. Risk of bias in observational studies was assessed using the Newcastle‐Ottawa Scale (NOS), while the Cochrane Risk of Bias Tool was used for randomized controlled trials (Table 1).

**Table 1:** Risk of bias table.

Study	Year	Study design	Bias assessment tool	Score/Assessment
Nguyen et al.	2000	Retrospective chart review	Newcastle‐Ottawa Scale	Low risk
Sadler et al.	2001	Cancer education program evaluation	Cochrane Risk of Bias Tool	Low risk
Nguyen et al.	2001	Pre‐/post‐intervention control group study	Cochrane Risk of Bias Tool	Low risk
Lin et al.	2002	Retrospective analysis	Newcastle‐Ottawa Scale	Low risk
Lin et al.	2002	Retrospective analysis	Newcastle‐Ottawa Scale	Low risk
Li et al.	2003	Retrospective cohort study	Newcastle‐Ottawa Scale	Low risk
McGarvey et al.	2003	Cross‐sectional survey study	Newcastle‐Ottawa Scale	Low risk
Yi & Luong	2005	Randomized controlled trial	Cochrane Risk of Bias Tool	Low risk
Ho et al.	2005	Mail survey	Newcastle‐Ottawa Scale	Moderate risk
Kandula et al.	2006	Cross‐sectional study	Newcastle‐Ottawa Scale	Low risk
Gomez et al.	2007	Cross‐sectional study	Newcastle‐Ottawa Scale	Low risk
Samuel et al.	2009	Chart review and questionnaire study	Newcastle‐Ottawa Scale	Low risk
Nguyen et al.	2009	Randomized Controlled Trial (RCT)	Cochrane Risk of Bias Tool	Moderate risk
Williams et al.	2009	Comparative tissue microarray study	Newcastle‐Ottawa Scale	Low risk
Ma et al.	2009	Cross‐sectional study	Newcastle‐Ottawa Scale	Low risk
Gomez et al.	2010	Population‐based study	Newcastle‐Ottawa Scale	Low risk
Le, MG	2011	Community‐based intervention study	Cochrane Risk of Bias Tool	Low risk
Nguyen et al.	2011	Qualitative study	CASP Qualitative Checklist	Moderate risk
Telli et al.	2011	Retrospective cohort study	Newcastle‐Ottawa Scale	Low risk
Nguyen et al.	2011	Cross‐sectional study	Cochrane Risk of Bias Tool	Low risk
Yi et al.	2011	Cross‐sectional study	Newcastle‐Ottawa Scale	Low risk
Nguyen & Belgrave	2011	CBPR (three‐phase pilot study)	Cochrane Risk of Bias Tool	Low risk
Yi et al.	2012	Retrospective analysis	Newcastle‐Ottawa Scale	Low risk
Gomez et al.	2012	Retrospective cohort study	Newcastle‐Ottawa Scale	Low risk
Ma et al.	2012	Cross‐sectional study	Newcastle‐Ottawa Scale	Low risk
Shaw et al.	2012	Mixed‐method (quantitative + qualitative)	Mixed‐methods Appraisal Tool	Moderate risk
Ryu et al.	2013	Population‐based study	Newcastle‐Ottawa Scale	Low risk
Nguyen AB & Belgrave FZ	2014	Community‐based participatory research	Cochrane Risk of Bias Tool	Low risk
Nguyen et al.	2014	Cross‐sectional study	Newcastle‐Ottawa Scale	Low risk
Nguyen & Clark	2014	Cross‐sectional study	Newcastle‐Ottawa Scale	Low risk
Radhakrishnan et al.	2015	Retrospective analysis	Newcastle‐Ottawa Scale	Low risk
Trinh et al.	2015	Retrospective cohort study	Newcastle‐Ottawa Scale	Low risk
Nguyen‐Truong et al.	2017	Quasi‐experimental design	Cochrane Risk of Bias Tool	Low risk
Nguyen et al.	2018	Cross‐sectional study	Newcastle‐Ottawa Scale	Low risk
Nguyen‐Truong et al.	2018	CBPR qualitative study	CASP Qualitative Checklist	Moderate risk
Shon & Townsend	2019	Retrospective cohort study	Newcastle‐Ottawa Scale	Low risk
Nguyen et al.	2020	Qualitative study	CASP Qualitative Checklist	Moderate risk
Thai et al.	2020	Patient navigator intervention study	Cochrane Risk of Bias Tool	Low risk
Medina et al.	2021	Retrospective cohort study	Newcastle‐Ottawa Scale	Low risk
Bock et al.	2023	Retrospective analysis	Newcastle‐Ottawa Scale	Low risk
Patel et al.	2023	Retrospective cohort study	Newcastle‐Ottawa Scale	Low risk

Given the heterogeneity in study designs, metrics, and outcomes, a narrative synthesis was conducted, organizing results into thematic categories: breast cancer screening behavior and barriers, breast cancer outcomes, interventions and education, molecular/genetic features, and broader disparities in access and care. Quantitative meta‐analysis was considered for outcomes with comparable measures, such as odds ratios for mammography screening or late‐stage diagnosis, but methodological and population variability precluded pooling of data. Instead, findings were synthesized qualitatively to identify patterns, gaps, and implications. This review exclusively included studies from primary databases and avoided reviews or secondary analyses to focus solely on original research. As all data were publicly available, ethical approval was not required.

## 3 Results

This systematic review includes 41 studies and 39,324 Vietnamese participants with a mean age of 48.15 years (SD = 7.48). The following themes were included in the narrative synthesis of these studies: breast cancer screening behavior and barriers, breast cancer outcomes, interventions and education, molecular/genetic features, and disparities to access and care (Table 2). In this population, social integration refers to the degree of connectedness within family and community networks, while instrumental support encompasses tangible assistance such as transportation or appointment scheduling. Acculturation describes the extent to which individuals adopt U.S. cultural norms, including language proficiency and familiarity with the healthcare system.

**Table 2:** Summary of included studies.

Study	Year	Study design	Total Ptcp.	Number of Viet. Ptcp.	Age (mean or median, years)	Breast cancer metric	Key findings of statistical analyses	Key findings in general
Nguyen et al.	2000	Retrospective chart review	952	468	Mean: 57 years	Mammogram utilization rates	Only 26% of eligible Vietnamese women aged ≥ 40 had mammograms, which was lower compared to other populations in Hawaii.	Vietnamese women had lower breast cancer screening rates than other ethnic groups in Hawaii. Education and insurance influenced screening rates.
Sadler et al.	2001	Cancer education program evaluation	275	275	Not reported	Mammography screening rates	Only 36% of women reported adequate breast cancer knowledge. Mammography rates were below recommended levels. 87% expressed interest in receiving more information.	Vietnamese American women had low levels of breast cancer knowledge and low adherence to mammography screening guidelines, highlighting the need for culturally targeted education programs.
Nguyen et al.	2001	Pre‐/post‐intervention control group study	1,595	789 (intervention: 384; control: 405)	Not reported	Screening (CBE and mammography)	The intervention had little effect on CBE and mammogram uptake in the general intervention population. However, women exposed to multiple intervention elements were significantly more likely to engage in screening behaviors.	A media‐led and neighborhood‐based intervention targeting Vietnamese women had mixed success. Exposure to intervention elements was key to influencing screening behaviors. Tailored outreach and evaluation methods are critical for effectiveness.
Lin et al.	2002	Retrospective analysis using SEER registry	280	280	Mean: 51.0 years	Tumor characteristics and surgery types	Vietnamese women were younger at diagnosis (mean age: 51 years) and more likely to receive mastectomy (61.1% for in situ/localized tumors) than other ethnic groups. The hospital of diagnosis partially explained these trends.	Vietnamese women showed higher mastectomy rates and younger age at diagnosis compared to other racial or ethnic groups. Further investigation is needed into cultural and systemic factors contributing to these trends.
Lin et al.	2002	Population‐based study using Greater Bay Area Cancer Registry data (SEER)	N/A (subset of registry data)	49 diagnosed cases	Mean: 51.0 years	Mastectomy rate, estrogen/progesterone receptor status	Vietnamese women were 80% more likely to have mastectomies compared to non‐Hispanic whites, even after adjusting for confounders like age and stage.	Vietnamese women have significantly younger diagnosis ages and higher rates of mastectomy due to cultural and hospital‐specific factors.
Li et al.	2003	Retrospective cohort study using SEER data	124,934	Included in API subgroup	Not reported	Stage at diagnosis, treatment adherence, survival rates	Vietnamese women had poorer survival rates compared to non‐Hispanic whites (adjusted HR > 1). However, Vietnamese women were 20% less likely to receive inappropriate therapy compared to other groups within the Asian subgroup.	Vietnamese women face disparities in survival outcomes and treatment adherence. Cultural and socioeconomic factors may contribute to these disparities. Tailored interventions are needed to improve outcomes for this group.
McGarvey et al.	2003	Cross‐sectional survey study	78	78	Not reported	Barriers and attitudes toward mammography	Vietnamese women perceived significant barriers to mammography, similar to Hispanic women. Barriers included cost, lack of knowledge, and cultural stigma.	Vietnamese and Hispanic women shared similar health beliefs and behaviors, but significant differences were observed among Cambodian women, emphasizing the need for subgroup‐specific strategies.
Yi & Luong	2005	Randomized controlled trial (intervention vs. control)	345	345	Mean: 55 years	Breast cancer knowledge and screening behaviors	Intervention group significantly improved in breast cancer knowledge (mean score increase: +4.2, *p* < 0.001) and increased breast self‐exam practices (+50.8%, *p* < 0.001) compared to control.	Apartment‐based education is effective for reaching underserved Vietnamese women and improving breast cancer screening knowledge and practices. Tailored, culturally sensitive interventions are critical for success.
Ho et al.	2005	Mail survey	209	209	Mean: 44.4 years	Use of BSE, MBE, and mammography	Predictors for screening included marital status, higher education, lack of barriers, family history of cancer, and older age.	Vietnamese women in Harris County had higher screening rates compared to other Vietnamese populations studied; barriers included lack of knowledge and access.
Kandula et al.	2006	Population‐based cross‐sectional study (CHIS 2001)	41,598	857	Reported age: 39.5 years	Mammography screening rates	After adjusting for access to care, Vietnamese women had a 72.7% mammography screening rate compared to 77.9% for non‐Hispanic whites. Language barriers and nativity significantly influenced screening behaviors.	Vietnamese women had relatively high mammography rates compared to other Asian subgroups but faced cultural and structural barriers that impacted overall cancer screening rates.
Gomez et al.	2007	Cross‐sectional study using CHIS data	1,521	226	Not reported	Mammography screening rates	72% of Vietnamese women without a prior Pap smear did not have a mammogram in the past two years. Single Vietnamese women were also at higher risk of non‐adherence to screening guidelines.	Vietnamese women faced higher disparities in mammography use compared to other Asian subgroups. Barriers included lack of Pap screening, single marital status, and lack of access to care, highlighting the need for targeted, culturally appropriate interventions.
Samuel et al.	2009	Chart review and questionnaire study	100	29	Mean: 62 years	Mammography screening rates	Vietnamese women had higher mammography screening rates compared to Somali and Cambodian women. Duration of U.S. residency positively correlated with screening rates for colorectal cancer but not breast cancer.	Vietnamese women were more likely to undergo breast cancer screening than Somali or Cambodian women, but cultural barriers and provider availability remained significant challenges.
Nguyen et al.	2009	Randomized Controlled Trial (RCT)	1,100	1,100	Mean: 57.3 years	Mammogram and Clinical Breast Exam (CBE) rates	Lay Health Worker Outreach (LHWO) combined with Media Education (ME) significantly increased mammography (OR 3.14) and CBE rates (OR 3.04) compared to ME alone.	LHWO combined with ME is an effective method to improve breast cancer screening rates in Vietnamese‐American women.
Williams et al.	2009	Comparative tissue microarray study	90	34	Mean: 53.3 years	Triple‐negative breast cancer (TNBC) marker expression and histology	Vietnamese women had smaller tumor sizes (3.2 cm vs. 4.7 cm) and lower rates of grade III tumors (62% vs. 84%). EGFR and P‐cadherin markers were underexpressed in Vietnamese women compared to the U.S. cohort, while CK8 was overexpressed.	Vietnamese TNBC showed less aggressive features compared to U.S. counterparts, with distinct marker expression patterns suggesting biological differences.
Ma et al.	2009	Cross‐sectional study	2011	362	Not reported	Mammogram screening rates among Asian subgroups	Screening rates varied significantly by subgroup; Vietnamese women reported screening rates ranging from 26% to 56% depending on various barriers.	Vietnamese women exhibited significant barriers to screening, including lack of health insurance and access to healthcare providers.
Gomez et al.	2010	Population‐based study (California Cancer Registry data)	20,747	1,584	Not reported	Breast cancer survival	Foreign‐born Vietnamese women had nearly four times higher mortality than US‐born Vietnamese women (HR = 3.9, 95% CI: 1.4–10.6). US‐born Vietnamese women had a lower risk of breast cancer mortality compared to all other Asian subgroups.	Survival differences among Asian subgroups were influenced by immigrant status, neighborhood SES, and other demographic factors. Vietnamese women showed stark survival disparities based on nativity.
Le, MG	2011	Community‐based intervention study (REACH 2010)	1,100	1,100	Mean: 56.7 years	Screening (mammography, CBE)	Higher social network integration significantly increased likelihood of receiving clinical breast exams (OR = 1.20, 95% CI: 1.07–1.33). Instrumental support was a significant predictor for mammography use (OR = 1.05, 95% CI: 1.02–1.08).	Vietnamese women reported lower screening rates compared to non‐Hispanic whites, with barriers linked to cultural factors, lack of insurance, and physician recommendations. Social support was critical in influencing screening behavior.
Nguyen et al.	2011	Qualitative study (focus groups & interviews)	110 (focus groups), 10 CBHNs, 15 providers	12	Not reported	Breast health navigation services	Vietnamese participants highlighted barriers such as limited English proficiency, fragmented healthcare systems, and lack of awareness about breast cancer. Community‐Based Health Navigators (CBHNs) provided critical logistical and emotional support.	CBHNs significantly improved screening access and patient‐provider communication through culturally tailored interventions. Training programs were recommended to standardize CBHN roles and increase their effectiveness.
Telli et al.	2011	Retrospective cohort study (2002–2007 CCR data)	89,009	663	Not reported	Breast cancer subtypes (HER2, TNBC)	Vietnamese women had a 29% frequency of HER2‐positive breast cancer, significantly higher than NH White women (19%). The odds ratio (OR) for HER2+ disease was 1.3 (95% CI: 1.1–1.6). Triple‐negative breast cancer frequency was 14%.	Vietnamese women had a distinct molecular subtype distribution compared to NH White women, with higher HER2‐positive and moderate triple‐negative rates. Age, stage, SES, and nativity influenced subtype distributions.
Nguyen et al.	2011	Quantitative, cross‐sectional study	111	111	Not reported	Screening efficacy and behavior	High levels of acculturation were associated with higher self‐efficacy for screening. Social extrinsic religiosity improved efficacy among less acculturated women. Intrinsic religiosity correlated with higher screening likelihood for highly acculturated women.	Acculturation plays a critical role in moderating the relationship between religiosity and cancer screening behavior among Vietnamese women. Tailored interventions should consider cultural and religious influences.
Yi et al.	2011	Cross‐sectional study	98	25	Mean: 56 years	Symptom distress and quality of life (QOL)	Vietnamese women reported significantly higher symptom distress scores (mean SDS = 35) compared to Chinese women (mean SDS = 20; *p* < 0.001). Vietnamese survivors had lower QOL scores, especially in physical, psychological, and social well‐being.	English proficiency was a significant predictor of symptom distress and QOL. Vietnamese survivors experienced greater symptom distress and lower QOL, highlighting the need for targeted support and culturally tailored interventions.
Nguyen & Belgrave	2011	Community‐Based Participatory Research (CBPR); three‐phase pilot study	Phase 1: 42 (19 men, 23 women), Phase 2: 70 women, Phase 3: 21 women	112	Mean: 41 years	Screening behaviors (Clinical Breast Exams and Pap tests), knowledge, self‐efficacy, and cultural factors affecting screening	Acculturation positively related to cancer screening knowledge (*r* = 0.57, *p* ≤ 0.01). Ethnic identity negatively related to knowledge (r = −0.45, *p* ≤ 0.01). Significant increases in cancer knowledge and self‐efficacy post‐intervention (*p* < 0.05).	Vietnamese women had low rates of breast and cervical cancer screening, influenced by lack of knowledge, acculturation, and cultural factors. The CBPR intervention improved screening knowledge, self‐efficacy, and intention to screen.
Yi et al.	2012	Retrospective analysis of SEER data (1988–2008)	658,691	2,071	Median: 51 years	Disease‐specific survival (DSS), tumor features	Vietnamese women had a 5‐year DSS rate of 89.6%. Younger age at diagnosis was noted (median age 51 years). Tumor stage and receptor status were significant predictors of survival.	Vietnamese women were among the youngest at diagnosis. Significant disparities in DSS existed compared to other Asian subgroups, emphasizing the need for tailored interventions in breast cancer care.
Gomez et al.	2012	Retrospective cohort study (1990–2007 CCR data)	20,987	1,444	Not reported	Mastectomy and BCS with radiation	Foreign‐born Vietnamese women had a 57.5% mastectomy rate, significantly higher than other Asian subgroups. Nativity, tumor size, and SES were critical determinants of surgical treatment.	Vietnamese women had the highest mastectomy rates among Asian subgroups, influenced by socioeconomic factors, tumor size, and nativity. Mastectomy rates decreased over time for all groups.
Ma et al.	2012	Cross‐sectional study using Sociocultural Health Behavior Model	682	145	Not reported	Mammography screening rates	Vietnamese women had a 12‐month mammography screening rate of 38%, lower than other Asian subgroups. Significant factors included acculturation (time in the U.S. < 15 years, limited English proficiency), enabling factors (insurance, regular physician), and family/social support.	Cultural, structural, and social barriers significantly influenced Vietnamese women's breast cancer screening rates. Addressing these barriers through tailored interventions can improve participation.
Shaw et al.	2012	Mixed‐method (quantitative + qualitative)	297	93	Not reported	Breast cancer screening (mammograms, self‐exams)	Positive attitudes toward cancer screenings were significantly correlated across types; negative attitudes were linked to lack of experience with screenings.	Social networks influence attitudes towards screenings, both positively and negatively. Medically underserved populations show barriers like fear, misinformation, and lack of access.
Ryu et al.	2013	Population‐based study using CHIS 2009	1,596 Asian immigrants	497	Reported age: 40+	Mammography screening rates	Vietnamese women had one of the highest rates of mammography screening (lifetime: 95.9%, recent: 82.8%) compared to other Asian subgroups. Acculturation and health insurance were significant predictors.	Screening disparities among Asian subgroups are influenced by acculturation, socio‐demographic factors, and health insurance. Vietnamese women had relatively high screening rates compared to Korean and Chinese immigrants.
Nguyen AB & Belgrave FZ	2014	CBPR with a 2×2 experimental design	103	103	Mean: 39 years	Breast cancer knowledge and screening	Intervention significantly improved breast cancer knowledge (*β* = 0.34, *p* = 0.002) and clinical breast exam rates (Nagelkerke R² = 0.36, *p* = 0.002). Ethnic identity moderated the intervention's effect, with lower ethnic identity predicting higher screening participation.	Culturally tailored CBPR interventions effectively improve cancer screening knowledge and behavior. Ethnic identity and collectivism play important roles in shaping screening attitudes and participation.
Nguyen et al.	2014	Cross‐sectional study using self‐reported data	100	100	Mean: 39.06 years	Screening (CBE and Pap test)	Acculturation positively associated with Pap test receipt. Masculine gender roles linked to higher screening self‐efficacy. Feminine gender roles had positive effects only in highly acculturated women.	Acculturation and gender roles significantly affect cancer screening behaviors. Tailored interventions addressing cultural barriers and leveraging community resources are essential for improving screening rates among Vietnamese women.
Nguyen & Clark	2014	Cross‐sectional study	111	111	Mean: 39.06 years	Self‐efficacy, attitudes, behavior	Higher levels of collectivism significantly predicted positive attitudes and higher self‐efficacy for cancer screening. Interaction between acculturation and collectivism showed varying effects on attitudes depending on collectivistic orientation.	Sociocultural factors like acculturation and collectivism significantly influence cancer screening attitudes and self‐efficacy. Tailored interventions addressing these cultural variables are essential for improving screening rates among Vietnamese women.
Radhakrishnan et al.	2015	Retrospective analysis of paired tumor/normal tissue samples	48	48	Not reported	Triple‐negative breast cancer (TNBC)	100% of normal paired samples showed biallelic IGF‐II expression. Tumors with biallelic IGF‐II gene expression exhibited higher levels of proIGF‐II and Survivin proteins.	LOI (loss of imprinting) and biallelic IGF‐II gene expression were associated with aggressive tumor phenotypes in Vietnamese TNBC patients.
Trinh et al.	2015	Retrospective cohort study using SEER database	776,498	3,549	Mean: 58.8 years	Cancer‐specific mortality rates and receipt of definitive treatment compared between Vietnamese, other Asian subgroups, and non‐Hispanic whites.	Vietnamese patients had better cancer‐specific mortality (CSM) rates for lung cancer compared to non‐Hispanic whites (HR = 0.88, 95% CI = 0.80‐0.96, *p* = 0.005). Differences in receipt of definitive treatment were observed across stages for various cancers.	Most Asian subgroups, including Vietnamese, had better cancer‐specific survival outcomes compared to non‐Hispanic whites. Sociocultural and genetic factors may play a role.
Nguyen‐Truong et al.	2017	One‐group, pre‐/post‐test, pilot, quasi‐experimental design	40	40	Reported age: ≥ 50 years	Mammogram completion and breast cancer knowledge	Significant increases in breast cancer knowledge, perceived susceptibility, and perceived benefits of mammography after the intervention. Mammogram completion rates improved post‐intervention.	The intervention was well‐accepted, feasible, and showed promising results in improving breast health education and mammogram completion among Vietnamese American immigrant women.
Nguyen et al.	2018	Cross‐sectional study	Not reported	Not reported	Not reported	Screening utilization	Limited English proficiency, lack of insurance, and cultural beliefs were significant barriers to breast cancer screening among Vietnamese American women.	Vietnamese American women were less likely to participate in routine breast cancer screening compared to other groups, emphasizing the need for culturally tailored health interventions.
Nguyen‐Truong et al.	2018	Community‐based participatory qualitative study	40	40	Not reported	Breast cancer beliefs and screening rates	Screening rates among Vietnamese American women were 64%, significantly below the national average (81.1%). Fear of pain, lack of knowledge, and cultural beliefs affected screening adherence.	Vietnamese American women face unique barriers to breast cancer screening, including fear, reliance on self‐detection, cultural beliefs in fate, and limited knowledge. Tailored interventions addressing these barriers are recommended.
Shon & Townsend	2019	Retrospective cohort study using survey data	3,710 (unweighted), 1,710,233 (weighted)	1,227 (weighted *n* = 405,000)	Not reported	Lifetime mammography rates	Vietnamese women had lower odds of never having a mammogram compared to Korean women.	Vietnamese women were among the groups with the lowest rates of never having a mammogram, but barriers included acculturation and health resource accessibility.
Nguyen et al.	2020	Qualitative study using focus groups	60	60	Median: 52 years	Screening (mammography and CBE)	Key barriers identified included language barriers, lack of insurance, cultural stigma, and lack of physician recommendation. Enabling factors included family support and community outreach programs.	Vietnamese women faced significant challenges in accessing breast cancer screening services due to cultural, systemic, and socioeconomic barriers. Tailored education and community interventions were suggested to improve participation.
Thai et al.	2020	Patient navigator intervention study	96	96	Mean: 62 years	Follow‐up care after abnormal mammogram	100% of participants attended follow‐up appointments within 3 months of receiving an abnormal mammogram. Psychosocial outcomes showed mixed results, with improvements in perceived control but increased anxiety and fear in some cases.	Patient navigator programs successfully improved follow‐up adherence but highlighted the need for culturally tailored interventions to address psychosocial outcomes among Vietnamese‐American women.
Medina et al.	2021	Retrospective cohort study (2012–2017)	260,914	4,257	Median: 45 years	Breast cancer mortality rates	Vietnamese women had significantly lower breast cancer mortality compared to NHW women, but liver and stomach cancer mortality were disproportionately higher. Socioeconomic status and hepatitis B prevalence were critical factors.	Vietnamese women face distinct cancer mortality patterns, including high liver and stomach cancer mortality. Breast cancer mortality was lower, indicating potential screening and early detection success in this group.
Bock et al.	2023	Retrospective analysis using cancer registry data	273,656 Asian, 18,491 NHPI	22,111	Not reported	Late‐stage breast cancer diagnosis	Late‐stage breast cancer diagnosis for Vietnamese individuals was 32.9%, with early‐stage diagnosis at 64.9%.	Lung cancer was the most common cancer, but breast cancer represented 13.9% of all new cancer diagnoses among Vietnamese individuals. Vietnamese individuals had higher rates of late‐stage diagnoses compared to other Southeast Asian subgroups.
Patel et al.	2023	Retrospective cohort study (NCDB 2004–2017)	1,670,528	663	Median: 61 years	Time to surgery (≥ 90 days)	Vietnamese women had a 17% higher odds of delayed surgery (OR: 1.17, 95% CI: 1.06–1.29, *p* < 0.01) compared to white women. Socioeconomic status, advanced stage, and nativity were significant predictors of delays.	Delays in surgery were more likely for Vietnamese women compared to white women, emphasizing disparities in care and the need for culturally tailored interventions to address barriers in timely treatment.

Abbreviations: BSE, Breast Self‐Examination; CBE, Clinical Breast Examination; CBHN, Community‐Based Health Navigator; CBPR, Community‐Based Participatory Research; CCR, California Cancer Registry; CHIS, California Health Interview Survey; CK8, Cytokeratin 8; DSS, Disease‐Specific Survival; EGFR, Epidermal Growth Factor Receptor; HR, Hazard Ratio; IGF‐II, Insulin‐Like Growth Factor II; IQR, Interquartile Range; LHWO, Lay Health Worker Outreach; LOI, Loss of Imprinting; MBE, Manual Breast Examination; ME, Media Education; NCDB, National Cancer Database; NHW, Non‐Hispanic White; OR, Odds Ratio; QOL, Quality of Life; RCT, Randomized Controlled Trial; REACH, Racial and Ethnic Approaches to Community Health; SEER, Surveillance, Epidemiology, and End Results Program; SES, Socioeconomic Status; SDS, Symptom Distress Scale; TNBC, Triple‐Negative Breast Cancer.

### 3.1 Breast Cancer Screening Behavior and Barriers

Breast cancer screening behavior among Vietnamese women is shaped by a complex interplay of social, cultural, and systemic factors, as evidenced by multiple studies. Social networks emerged as critical influencers in Le (2011), where increased social integration significantly enhanced clinical breast exam (CBE) uptake (OR = 1.20, 95% CI: 1.07–1.33) and instrumental support predicted mammography use (OR = 1.05, 95% CI: 1.02–1.08) [9]. Similarly, Shaw et al. (2012) found social connections influence cancer screening, with family and friends encouraging screening through shared positive experiences, while misinformation, such as beliefs that screenings are unnecessary without symptoms, discouraged participation [10]. Furthermore, several studies identified systemic barriers to screening. Nguyen et al. (2018) found that limited English proficiency, lack of insurance, and cultural beliefs significantly deterred Vietnamese American women from participating in routine screening programs [11]. Similarly, McGarvey et al. (2003) reported that cost, lack of knowledge, and cultural stigma were perceived as significant barriers, paralleling findings in Sadler et al. (2001), where Vietnamese women displayed low levels of breast cancer knowledge, leading to low adherence to mammography guidelines [12, 13]. Shon et al. (2019), Ho et al. (2005), Ma et al. (2009) and Ma et al. (2012) corroborated these findings [14, 15, 16, 17].

Cultural factors, such as collectivism and acculturation, play a crucial role in shaping cancer screening behaviors. Collectivism, which emphasizes the importance of family and community over individual concerns, fosters greater social support and encouragement for preventive health behaviors. In Vietnamese communities, this often manifests as family members urging women to undergo screening or accompanying them to appointments [18]. Acculturation, the process of adapting to the norms and practices of a new culture, similarly influences screening attitudes. Higher levels of acculturation are associated with increased familiarity with the U.S. healthcare system, improved English proficiency, and greater self‐efficacy in seeking preventive care, ultimately contributing to higher screening participation [18]. Nguyen AB and Belgrave (2014) similarly reported that ethnic identity moderated cancer screening behaviors, with culturally tailored community‐based participatory research (CBPR) interventions effectively improving screening knowledge and participation [19]. Education and outreach programs demonstrated mixed success in improving screening rates. Nguyen et al. (2001) noted that media‐led and neighborhood‐based interventions had limited effects overall but significantly improved screening behaviors when participants were exposed to multiple intervention elements [20]. In contrast, Nguyen‐Truong et al. (2018) documented that Vietnamese‐American women's screening rates (64%) in their study fell below the national average, with fear, cultural beliefs, and lack of knowledge being primary barriers [21].

Vietnamese women face significant barriers related to language and nativity. Ryu et al. (2013) observed that higher screening rates often reflect participation among more acculturated individuals, while less acculturated women experience difficulty navigating healthcare systems due to limited English proficiency, unfamiliarity with preventive care practices, and mistrust stemming from cultural differences [22]. Specifically, Vietnamese women with limited English proficiency were 43% less likely to undergo mammography screening compared to their English‐proficient counterparts (OR = 0.57, 95% CI: 0.42–0.78) [11]. Kandula et al. (2006) similarly reported that Vietnamese women with less than 10 years of U.S. residency were significantly less likely to engage in mammography screening compared to those with longer residency durations (72.7% vs. 82.5%; *p* < 0.01) [23]. These findings suggest that the reported screening success may mask underlying disparities, particularly among recent immigrants and those with lower acculturation levels.

**Figure 2:**
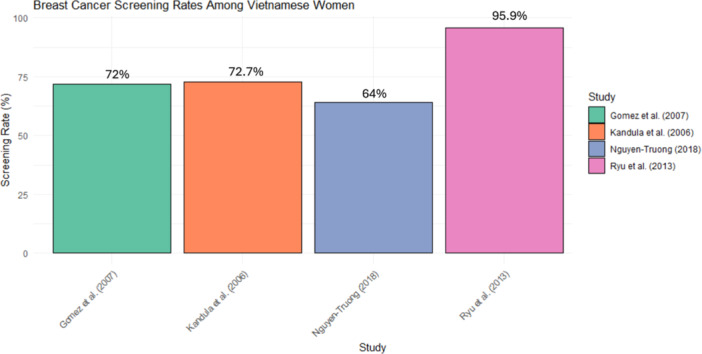
Bar plot of breast cancer screening rates among Vietnamese women.

Lastly, disparities in mammography utilization were underscored by multiple studies. Gomez et al. (2007) reported that Vietnamese women who had not undergone prior Pap smears were less likely to have mammograms, while single marital status further increased the risk of non‐adherence [24]. Similarly, Ly I. Nguyen et al. (2000) found that Vietnamese women in Hawaii exhibited lower mammography rates (26%) compared to other ethnic groups, with the CDC reporting the U.S. national average of mammography at 81%, emphasizing the need for interventions tailored to address specific cultural and systemic challenges [25]. Figure 2 illustrates the variability in screening rates across studies reporting mammography utilization among Vietnamese women. Percentages ranged from 26% in Nguyen et al. (2000) to 83% in Ryu et al. (2013), with higher rates generally observed in more acculturated subgroups. Late‐stage breast cancer diagnosis is also a persistent issue. According to Bock et al. (2023), 33% of breast cancer diagnoses among Vietnamese individuals occurred at a late stage, which compares to 30% in non‐Hispanic white (NHW) women, indicating challenges in early detection and intervention [26].

### 3.2 Breast Cancer Outcomes

The outcomes of breast cancer among Vietnamese women reveal substantial disparities in survival rates, tumor characteristics, and treatment patterns when compared to other ethnic groups. Survival outcomes for Vietnamese women are significantly influenced by nativity and socioeconomic factors. Gomez et al. (2010) highlighted that foreign‐born Vietnamese women exhibited nearly four times higher mortality than U.S.‐born Vietnamese women (HR = 3.9, 95% CI: 1.4–10.6) [27]. Similarly, Li et al. (2003) reported poorer survival rates for Vietnamese women compared to NHW women, despite a lower likelihood of receiving inappropriate therapy [28]. Conversely, Medina et al. (2021) observed that Vietnamese women had lower breast cancer mortality rates compared to NHW women (12.4 per 100,000 vs. 20.2 per 100,000), potentially reflecting the success of screening and early detection efforts in some subpopulations [29].

Disease‐specific survival (DSS) data further underscore these disparities. Yi et al. (2012) found a 5‐year DSS rate of 89.6% for Vietnamese women, noting that younger age at diagnosis (median: 51 years) and tumor stage were significant predictors of survival [30]. This compares to a 5‐year DSS for U.S. breast cancer overall of 92%. This aligns with findings by Lin et al. (2002), who reported that Vietnamese women were diagnosed at a younger age (mean: 51 years) compared to other racial groups and exhibited higher mastectomy rates (61.1%) for in situ or localized tumors in comparison to the national U.S. rates of 35–40% [31]. Moreover, treatment disparities are evident, particularly in surgical interventions. Nguyen et al. (2009) and Lin et al. (2002) both highlighted significantly higher mastectomy rates among Vietnamese women, which were often influenced by nativity, tumor stage, and cultural factors [31, 32]. Patel et al. (2023) revealed that Vietnamese women had a 17% higher odds of delayed surgery (OR = 1.17, 95% CI: 1.06–1.29), emphasizing disparities in timely access to care [33].

### 3.3 Interventions & Education

Efforts to improve breast cancer awareness and screening behaviors among Vietnamese women have focused on culturally tailored interventions and education programs, with significant success in enhancing knowledge, attitudes, and adherence to screening recommendations. For example, Nguyen‐Truong et al. (2017) demonstrated the feasibility and effectiveness of a multicomponent breast health education intervention targeting Vietnamese American immigrant women aged ≥ 50 years [34]. This pilot study reported a significant improvement in breast cancer knowledge (mean pre‐test score: 0.33 ± 1.37 vs. post‐test: 0.63 ± 0.53, *t*(39) = –14.72, *p* < 0.001) and perceived susceptibility to breast cancer (mean pre‐test: 2.87 ± 0.83 vs. post‐test: 3.32 ± 0.70, *t*(39) = –2.69, *p* < 0.05). Moreover, the intervention successfully increased mammogram completion rates, with 30 out of 39 participants (77%) undergoing mammography within 12 weeks of the intervention from a baseline of 0%, highlighting the importance of culturally relevant, comprehensive education programs in addressing disparities in screening behaviors [34].

Community‐based participatory research (CBPR) has been instrumental in addressing cultural barriers to screening. Nguyen and Belgrave (2014) evaluated a CBPR intervention that emphasized ethnic identity and collectivism to enhance breast cancer screening knowledge and behavior [19]. The intervention significantly improved clinical breast exam rates (Nagelkerke R² = 0.36, *p* = 0.002) and highlighted how cultural values influence screening attitudes and participation [19]. Similarly, Nguyen, Belgrave, and Sholley (2011) conducted a three‐phase CBPR study that resulted in marked improvements in cancer knowledge and self‐efficacy among participants [35]. The intervention's emphasis on culturally tailored outreach was key to its success.

Thai et al. (2020) assessed the impact of a patient navigator program designed for Vietnamese‐American women with abnormal mammograms [36]. The program achieved 100% adherence to follow‐up appointments within 3 months of receiving abnormal results, underscoring the critical role of navigators in addressing logistical and emotional barriers. However, mixed psychosocial outcomes, such as increased anxiety in some participants, pointed to the need for further refinement of intervention strategies.

Apartment‐based education programs have also proven effective for underserved Vietnamese women. Yi and Luong (2005) conducted a randomized controlled trial evaluating a program aimed at increasing breast cancer knowledge and breast self‐exam (BSE) practices among low‐income Vietnamese women [37]. The intervention group showed significant improvements in knowledge (mean score increase: +4.2, *p* < 0.001) and a 50.8% increase in BSE practices (*p* < 0.001). The program's localized and culturally sensitive approach made it particularly successful in reaching marginalized populations.

Finally, Nguyen and Clark (2014) examined the interplay between cultural barriers, acculturation, and tailored interventions in cancer screening behaviors [18]. The study found that addressing collectivism and cultural norms, while leveraging community resources, significantly influenced participants’ screening self‐efficacy and attitudes. These findings emphasize the need for interventions that go beyond general health education to address sociocultural dynamics specific to Vietnamese women.

### 3.4 Disparities in Access & Care

Several studies highlight disparities in access to breast cancer screening, diagnosis, and care among Vietnamese women, emphasizing structural and cultural barriers that hinder equitable healthcare outcomes. Gomez et al. (2007) examined disparities in mammography use among Asian women in California, with a specific focus on Vietnamese women [38]. The study found that Vietnamese women who had not undergone a Pap smear were significantly less likely to have a mammogram in the past 2 years (72% non‐adherence). Single marital status and lack of access to care further exacerbated disparities in mammography adherence, suggesting that broader issues in women's healthcare access cascade across multiple cancer screening behaviors. Trinh et al. (2015) assessed cancer‐specific mortality across stages and treatment receipt among Asian American subgroups [39]. Disparities in the receipt of definitive treatment, including curative interventions like surgery, radiation, or systemic therapy, were evident across various breast cancer stages. This gap in care points to systemic barriers, such as healthcare infrastructure and socioeconomic constraints, that impact the timely diagnosis and treatment of cancer in Vietnamese populations.

### 3.5 Molecular/Genetic Features

Studies exploring the molecular and genetic features of breast cancer in Vietnamese populations have provided valuable insights into tumor biology and subtype‐specific characteristics, highlighting unique patterns that may influence prognosis and treatment strategies. Radhakrishnan et al. (2015) examined the role of IGF‐II expression in triple‐negative breast cancer (TNBC) among Vietnamese patients [40]. The study found that all paired normal tissue samples exhibited biallelic IGF‐II expression, while tumors with biallelic IGF‐II gene expression showed elevated levels of proIGF‐II and Survivin proteins. These findings linked the loss of imprinting (LOI) and biallelic IGF‐II expression with more aggressive TNBC phenotypes, underscoring the potential prognostic value of IGF‐II in this population. In addition, molecular characteristics of tumors vary among Vietnamese women. Telli et al. (2011) documented a 29% frequency of HER2‐positive breast cancer among Vietnamese women, significantly higher than NHW women (19%; OR = 1.3, 95% CI: 1.1–1.6) [41]. The study also identified a moderate frequency of triple‐negative breast cancer (TNBC) at 14%. Williams et al. (2009) investigated TNBC in Vietnamese women compared to U.S. counterparts using tissue microarray analysis [42]. Vietnamese TNBC cases were characterized by smaller tumor sizes (mean: 3.2 cm vs. 4.7 cm) and lower rates of grade III tumors (62% vs. 84%). Molecular marker expression also differed, with underexpression of EGFR and P‐cadherin and overexpression of CK8 in Vietnamese women. These differences suggest a less aggressive tumor phenotype in Vietnamese TNBC cases and point to distinct biological pathways underlying tumor development in this group.

## 4 Discussion

This systematic review is the first to comprehensively synthesize evidence on the unique disparities in breast cancer screening, diagnosis, treatment, and outcomes among Vietnamese‐American women, highlighting the combined influence of sociocultural, systemic, and molecular factors on care access and health outcomes.

The breast cancer screening behaviors of Vietnamese women are shaped by a complex interplay of cultural, systemic, and structural factors. Social networks, for instance, serve as both facilitators and barriers to screening uptake, depending on the nature and influence of these relationships. While social support has been shown to positively impact engagement with clinical breast exams (CBEs) and mammography, misinformation within these networks can perpetuate fear and misconceptions about cancer screening [9, 10]. This highlights the need for community‐driven educational campaigns that leverage trusted social networks to promote accurate information and encourage screening participation. Additionally, language barriers, lack of insurance, and cultural stigma remain persistent obstacles, particularly for recently immigrated populations [11, 12]. Interventions must move beyond simple language translation to include culturally congruent health messaging that resonates with the values and beliefs of Vietnamese women. Health systems should prioritize collaborations with community leaders to build trust and improve the effectiveness of outreach programs.

Additionally, disparities in breast cancer outcomes underscore the intersection of biological, structural, and socioeconomic factors. Late‐stage diagnosis remains a persistent issue, with 32.9% of Vietnamese breast cancer cases identified at an advanced stage [26]. Mortality rates are also disproportionately higher among foreign‐born Vietnamese women compared to their U.S.‐born counterparts, highlighting the influence of immigration‐related factors, including healthcare access, cultural perceptions of cancer, and health literacy [27]. Programs that extend beyond hospital‐based care to community settings may offer more accessible pathways for early detection and treatment adherence, especially for recently immigrated individuals.

Intervention studies demonstrate the potential effectiveness of culturally tailored programs to improve screening rates, though the heterogeneity in outcomes suggests that these programs require ongoing evaluation and adaptation. CBPR interventions have yielded promising results by integrating cultural norms like collectivism and familial decision‐making into educational materials [19, 35]. However, these programs are resource‐intensive and require context‐specific modifications to maintain engagement across diverse Vietnamese communities. Patient navigation programs have also demonstrated substantial success in reducing follow‐up loss after abnormal mammograms, but scaling these interventions requires sufficient resources and staffing [36]. Expanding the availability of navigators, particularly those with shared linguistic and cultural backgrounds, may help mitigate healthcare system navigation barriers.

Access to care remains a fundamental challenge for Vietnamese women, with linguistic, socioeconomic, and geographic barriers contributing to delayed diagnoses and suboptimal treatment outcomes. Language barriers have consistently been associated with lower screening rates and increased mortality risk [11, 30]. Geographic disparities are equally significant, with only 10% of cancer care facilities in Southern California offering Vietnamese‐language services [43, 44]. Additionally, Vietnamese women with lower acculturation levels are less likely to participate in screening and may delay follow‐up care after abnormal findings [24, 45]. Expanding access to culturally and linguistically tailored services, particularly in areas with large Vietnamese communities, should be a priority for policymakers [46, 47, 48]. Furthermore, structural interventions, such as insurance expansions and partnerships with community organizations, could help bridge gaps in care access [49]. Lastly, Telli et al. (2011) and Williams et al. (2009) found potential ethnic‐specific variations in tumor biology, which may have implications for treatment strategies [41, 42]. However, the current body of molecular research remains limited in scope, underscoring the need for further investigation into potential pharmacogenomic differences that could guide personalized care approaches.

### 4.1 Limitations

This systematic review has limitations. First, there were a limited number of studies focusing specifically on Vietnamese women and breast cancer. Despite a comprehensive search across multiple databases, relatively few studies provided disaggregated data for this population, particularly in areas such as molecular characteristics, treatment outcomes, and the effectiveness of interventions. Second, the heterogeneity in study designs, sample sizes, and outcome definitions limited the ability to perform a meta‐analysis. Variability in data sources, including self‐reported screening behaviors and registry data, introduces potential recall, reporting, and selection biases. Geographically, most studies focused on Vietnamese women in the United States, particularly in California and Texas, limiting generalizability to other regions. Few studies disaggregated findings by nativity or immigration status, despite their known influence on screening behaviors and outcomes. Future research should use standardized measures, include diverse populations, and incorporate longitudinal analyses to better understand these disparities.

## 5 Conclusion

Vietnamese women face unique challenges in breast cancer prevention and care, shaped by sociocultural, systemic, and biological factors. Disparities in screening utilization, late‐stage diagnoses, and access to timely care persist, driven by barriers such as limited English proficiency, cultural beliefs, and structural inequities in healthcare systems. Distinct molecular and genetic characteristics of breast cancer in this population further highlight the need for tailored diagnostic and therapeutic strategies. While community‐based interventions, such as patient navigation and culturally sensitive education programs, have demonstrated promise in improving knowledge and adherence to screening, these efforts must be expanded and integrated into broader healthcare policies. Addressing these challenges requires a comprehensive approach that combines culturally tailored education, improved access to linguistically competent care, and ongoing research into population‐specific tumor biology. By bridging these gaps, healthcare systems can promote equitable breast cancer outcomes for Vietnamese women and other underserved populations.

## Conflicts of Interest

The authors declare no conflicts of interest.

## Synopsis

This systematic review examines disparities in breast cancer screening, diagnosis, and outcomes among Vietnamese American women. Analyzing 41 studies with 39,324 participants, the review identifies sociocultural, linguistic, and systemic barriers that contribute to lower screening rates, later‐stage diagnoses, and distinct molecular tumor characteristics. Findings underscore the need for culturally tailored interventions, improved healthcare access, and further research into population‐specific tumor biology.

## Data Availability

The data that support the findings of this study are available in Google at https://www.google.com/. These data were derived from the following resources available in the public domain: ‐ PubMed, https://pubmed.ncbi.nlm.nih.gov/ ‐ Scopus, https://www.elsevier.com/products/scopus ‐ Embase, https://www.elsevier.com/products/embase.
